# Revealing Molecular-Scale Structural Changes in Polymer Nanocomposites during Thermo-Oxidative Degradation Using Evolved Gas Analysis with High-Resolution Time-of-Flight Mass Spectrometry Combined with Principal Component Analysis and Kendrick Mass Defect Analysis

**DOI:** 10.1021/acs.analchem.3c05269

**Published:** 2024-01-30

**Authors:** Ryota Watanabe, Sayaka Nakamura, Aki Sugahara, Mayumi Kishi, Hiroaki Sato, Hideaki Hagihara, Hideyuki Shinzawa

**Affiliations:** † Research Institute for Sustainable Chemistry, National Institute of Advanced Industrial Science and Technology (AIST), 1-1-1 Higashi, Tsukuba 305-8565, Japan

## Abstract

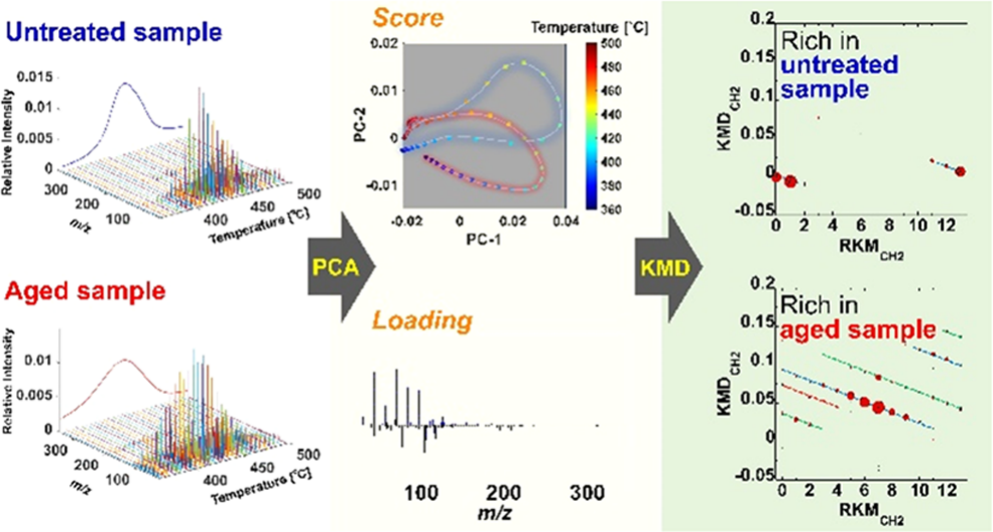

This study introduces a novel method that utilizes evolved gas analysis with time-of-flight mass spectrometry (EGA-TOFMS) coupled with principal component analysis (PCA) and Kendrick mass defect (KMD) analysis, called EGA-PCA-KMD, to analyze complex structural changes in polymer materials during thermo-oxidative degradation. While EGA-TOFMS captures exact mass data related to the degradation components in the temperature-dependent mass spectra of the evolved products, numerous high-resolution mass spectra with large amounts of ion signals and varying intensities provide challenges for interpretation. To address this, we employed mathematical decomposition through PCA to selectively extract information about the ion series specific to the products that evolved from the degradation components. Additionally, KMD analysis was applied to the attribution of the exact mass signals extracted from the PCA, which categorizes and visualizes depending on the molecular compositions in a two-dimensional plot. The complex structural changes of the triblock copolymer thermoplastic elastomer and its nanocomposites containing nanodiamonds during thermo-oxidative degradation were elucidated using EGA-PCA-KMD to demonstrate the effectiveness of this characterization technique for polymer degradation. Furthermore, it is revealed that the formation of rigid matrix–filler interfacial interaction via the π–π stacking and chemical bonds in the nanocomposites contributes to improvement in the stability toward thermo-oxidative degradation. Our results highlight the benefits of EGA-PCA-KMD and provide valuable insights into polymer degradation.

Thermo-oxidative degradation is regarded as a critical issue accompanied by sustainable growth in the industrial use of polymer materials. Additives such as antioxidants are essential components of polymer materials for long-term use because they suppress thermo-oxidative degradation.^1−3^ Over the past few years, nanocarbon fillers such as carbon nanotubes^4−8^ and graphene^9,10^ have attracted much attention as polymer antioxidants because of their radical-scavenging ability. To suppress degradation in material design, it is crucial to better understand the degradation mechanism of polymer materials. Therefore, there is a demand for techniques that can analyze the molecular-scale structures of degradation components with high sensitivity and accuracy.

Evolved gas analysis (EGA) is a highly effective tool for elucidating the structure and state of polymer materials by analyzing the temperature-dependent mass spectra of products evolved from the materials during temperature ramping.^11−17^ In EGA, improving the resolution of mass spectra is ideal for further enhancing the accuracy of estimating the molecular composition of the evolved products. The evolution of modern time-of-flight mass spectrometry has spurred a new trend in analytical data processing in recent years, owing to the acquisition of high-resolution mass spectra with high sensitivity. It is expected that EGA with time-of-flight mass spectrometry (EGA-TOFMS) can provide insights into the complex structural changes of polymer materials arising from thermo-oxidative degradation. However, the interpretation of high-resolution mass spectra dominated by many peaks is already a laborious task; therefore, the development of such vast amounts of data is a major obstacle to the conversion of experimental knowledge into valid conclusions. Herein, we propose a data mining technique, a combination of principal component analysis (PCA) and Kendrick mass defect (KMD) analysis, for temperature-dependent high-resolution mass spectra collected by EGA-TOFMS.

PCA is a mathematical tool used for the decomposition of two-way data into an orthogonal set of dominant factors known as eigenvectors.^18−22^ PCA results in the generation of two matrices, called scores and loadings, which represent all features distributed throughout the data set. When applied to EGA-TOFMS data, such as the analysis of polymer samples before and after aging, PCA can play a crucial role in extracting key mass information regarding the specific evolved products originating from degradation components. It can be an important task to attribute the specific mass data represented by PCA to understand the degradation behavior although the attribution of numerous exact mass peaks is extremely time-consuming. In this case, KMD analysis, which is a method for the categorization and visualization of the exact mass signals based on molecular compositions in a two-dimensional plot,^23−29^ is quite useful for comprehensively attributing the ion series specific to the pyrolysis of degradation products, as revealed by PCA.

In this study, the EGA-TOFMS technique coupled with PCA and KMD analyses for data mining, called EGA-PCA-KMD, was applied to examine the complex structural changes caused by thermo-oxidative degradation in a triblock copolymer thermoplastic elastomer and its composites containing nanodiamonds. The objective of this study is to demonstrate that EGA-PCA-KMD enables a precise understanding of the detailed structure and state of the degraded products. Furthermore, it provides insights into the influence of nanodiamonds on the progression of polymer degradation. The composites under investigation were fabricated by mixing a maleic anhydride-grafted styrene–ethylene butylene–styrene triblock copolymer (MASEBS) as a matrix with two types of nanodiamonds: one with surface modification with amino groups (AmND) and the other without (ND). In the case of the AmND and MASEBS composites, interfacial bonding was achieved through the generation of maleamide groups between the amino groups and maleic anhydride groups, as depicted in Figure S1. This process is similar to that of MASEBS composites containing silica spheres modified with amino groups.^30^ This study explores the effects of these interfacial structures on the radical trapping efficiency of nanodiamonds to suppress thermo-oxidative degradation.

## Experimental Section

### Materials

ND and AmND were purchased from Tokyo Chemical Industry. Field-emission scanning electron microscopy (FE-SEM) images of ND and AmNDs, with a size of 10 nm, are shown in Figure S2. The relative surface areas of ND and AmND, which are estimated by nitrogen adsorption–desorption isotherms, are 253 and 248 m^2^/g, respectively, indicating that the surface area is almost unchanged by surface modification. Carbon compositions, such as the D band with sp^3^ hybrid carbon and the G band with sp^2^ hybrid structures on ND and AmND, were examined by Raman spectrometry (Figure S3). MASEBS pellets containing 2 wt % maleic anhydride were purchased from Sigma-Aldrich.

### Sample Preparation

ND/MASEBS and AmND/MASEBS were fabricated by mixing 30 wt % fillers in 15 mL of a toluene solution containing 2.7 g of MASEBS at room temperature using magnetic stirring. Dried composite samples were obtained by evaporating toluene overnight at room temperature. Sample sheets (50 mm × 50 mm × 0.5 mm) for EGA-TOFMS were prepared using 2 g of the dried composites by hot pressing at 200 °C under 5 MPa for 3 min and then under 10 MPa for 10 min, using a Naflon sheet (Nichias), a stainless steel window frame of 0.5 mm in thickness, and stainless steel plates. The hot-pressed samples were then quickly quenched to room temperature. The aging treatment of the sample sheets was performed in an oven at 180 °C under an air atmosphere.

### EGA-TOFMS

The EGA-TOFMS system comprised a temperature-programmable microfurnace pyrolyzer (PY-3030D; Frontier Lab, Japan), gas chromatograph (7890 B; Agilent Technologies), and time-of-flight mass spectrometer (JMST200, JEOL, Japan). A sample weight (approximately 0.1 mg, sufficiently small to achieve instant thermodynamic equilibrium during programmed heating) was used for the EGA-TOFMS measurements. The sample was placed in a deactivated stainless steel sample cup and heated in a pyrolyzer from 100 to 700 °C at a heating rate of 10 °C min^–1^ under a helium atmosphere. A proportion of the flow (1 mL min^–1^) reduced by a GC splitter (50:1) was continuously introduced into the MS via a deactivated fused-silica column (2.5 m × 0.25 mm id, Agilent Technologies). The column was maintained at 300 °C in the GC oven to prevent the condensation of less volatile products in the capillary. The interface and ion source temperatures were set at 280 and 250 °C, respectively. For the MS measurements, electron ionization (EI) was performed with an operating mass range of *m*/*z* 30–800 and a recording interval of 0.5 s. Perfluorotributylamine (PFTBA) was used to tune the mass spectrometer. The peak resolution of ∼10,000 for *m*/*z* 501.970 was adjusted. The evolution profiles of the products were observed in the total ion current (TIC) and extracted ion monitoring (EIM) modes. The TIC mode represents the added intensities of all mass spectral peaks. A specific mass was extracted from all of the mass spectral peaks in the EIM mode.

## Results and Discussion

### Methods

#### PCA

In this paper, boldface capital letters are used to represent matrices, and the superscript “*t*” indicates the transposition of a matrix. A series of exact mass data for the evolved products were collected using EGA-TOFMS. The spectra can be represented as matrix ***X*** with *m* × *n* dimensions, as expressed in eq 1.
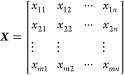
1where *m* is the number of spectra collected at different periods and *n* is the number of data points along the *m*/*z* axis. By applying singular value decomposition (SVD), matrix ***X*** of the mass spectra can be expressed as the product of two matrices **T** and **P** and a residual matrix ***E*** as eq 2.^18−22^
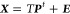
2where ***T*** is the *m* × *r* PCA score matrix and ***P*** is the *n* × *r* PCA loading matrix. Rank *r* represents the number of principal components (PCs) that correspond to a significant portion of the information distributed over the mass spectral data ***X***. Matrix ***E*** is the residual portion of the original data, which is not accounted for by the first *r* PCs used for data representation. ***T*** contains abstract information concerning the temperature-induced variation in the mass spectra of the evolved products, and ***P*** represents an important variable that provides chemically meaningful interpretations of the patterns observed in ***T***. A series of PCA were performed using the MATLAB version R2022b and Statistics and Machine Learning Toolbox (MathWorks).

#### KMD Analysis

KMD analysis highlights the differences between hydrocarbon ions and other structures in a two-dimensional plot. The exact mass data were converted to Kendrick masses (KM) using eq 3.^23,24^
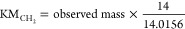
3KM_CH_2__ is KM when CH_2_ is set as the base unit. The unified atomic mass of CH_2_ is 14.0156, as defined by the International Union of Pure and Applied Chemistry (IUPAC). The KM_CH_2__ of each peak was rounded off to obtain integer KM values (nominal KM_CH_2__ and NKM_CH_2__). The Kendrick mass defect value (KMD_CH_2__), which is the difference between NMK_CH_2__ and KM_CH_2__, was calculated using eq 4.
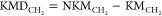
4NKM_CH_2__ and KMD_CH_2__ are plotted on the *x*- and *y*-axes, respectively. The distribution of each component was expressed in a bubble chart format.

The remaining KM (RKM) plots were used to search for components with hydrocarbon chains but different chemical structures (different functional groups, oxidation, unsaturated bonds, etc.) for CH_2_. The RKM value is expressed by the following equation using a modulo function, as given by eq 5.^25^
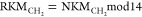
5where RKM_CH_2__ is the RKM when 14 (the integer mass of CH_2_) is set as the divisor. The RKM plot represents RKM as a function of NKM. A series of KMD analyses were performed using msRepeatFinder software (ver. 5.001, JEOL).

#### EGA-TOFMS Coupled with PCA and KMD

**Figure 1:**
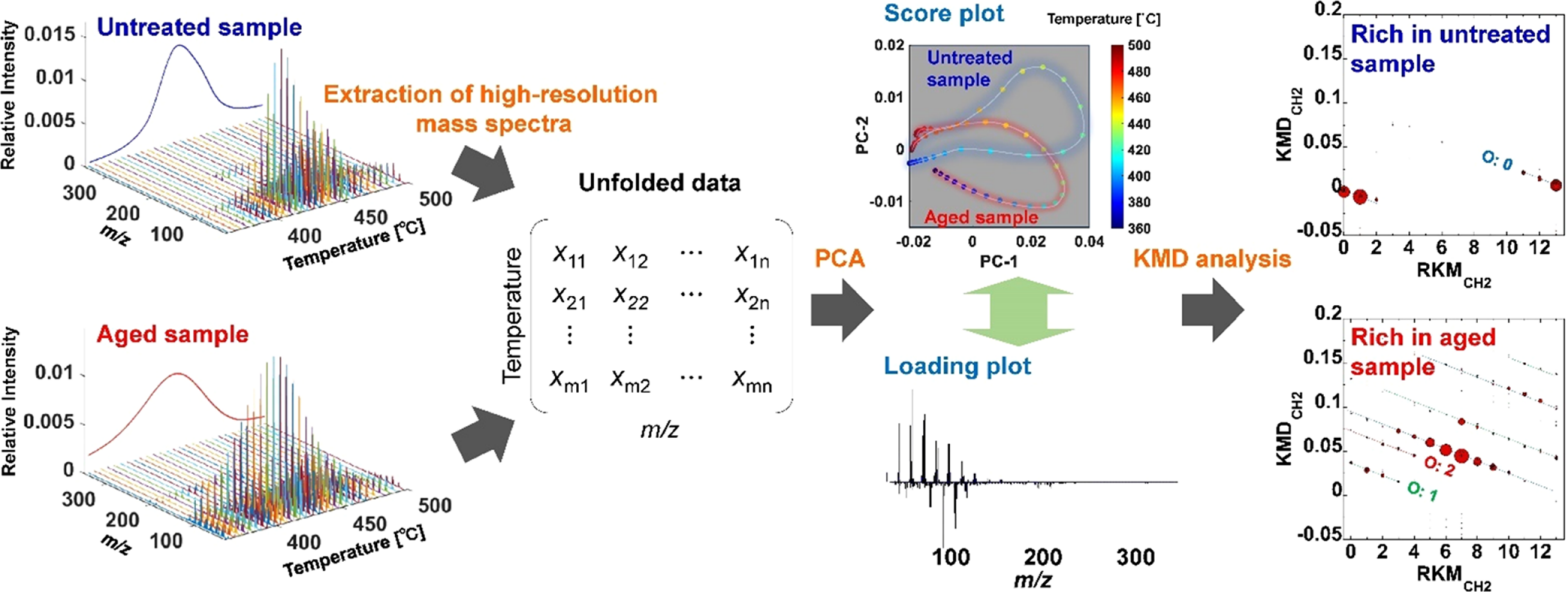
Elucidation of ion series specific to evolved products from untreated and aged samples by EGA-TOFMS combination with PCA and KMD analysis.

Figure 1 illustrates the PCA results of the three-way EGA-TOFMS data array, in which the mass-to-charge ratio (*m*/*z*), temperature, and presence or absence of aging treatment were obtained via unfolding. Unfolding is a technique based on the systematic concatenation of data segments to convert three-way data with two perturbations into a new linear data format with only one combined coordinate.^16^ The unfolding results in a two-way data array that can be subjected to conventional PCA to generate Score ***T*** and Load ***P*** matrices. The spectral changes caused by the aging treatment in the polymer system can be elucidated by exploring the patterns that appear in the plots of these scores. The first and second PCs, denoted PC-1 and PC-2, respectively, were two significant abstract components of the mass spectral series. The scores were projected as temperature-dependent trajectories on the PC-1/PC-2 plane. A score plot was constructed to visualize the degradation states of the polymer materials. The loadings of PC-1 and PC-2 are representative signals of the data set used in the PCA, which provides a chemical interpretation of patterns observed in the score plot. Finally, KMD analysis was applied to attribute the loadings comprehensively, revealing the molecular formulas of the characteristic ions closely associated with the patterns observed in the score plots.

### EGA-TOFMS

**Figure 2:**
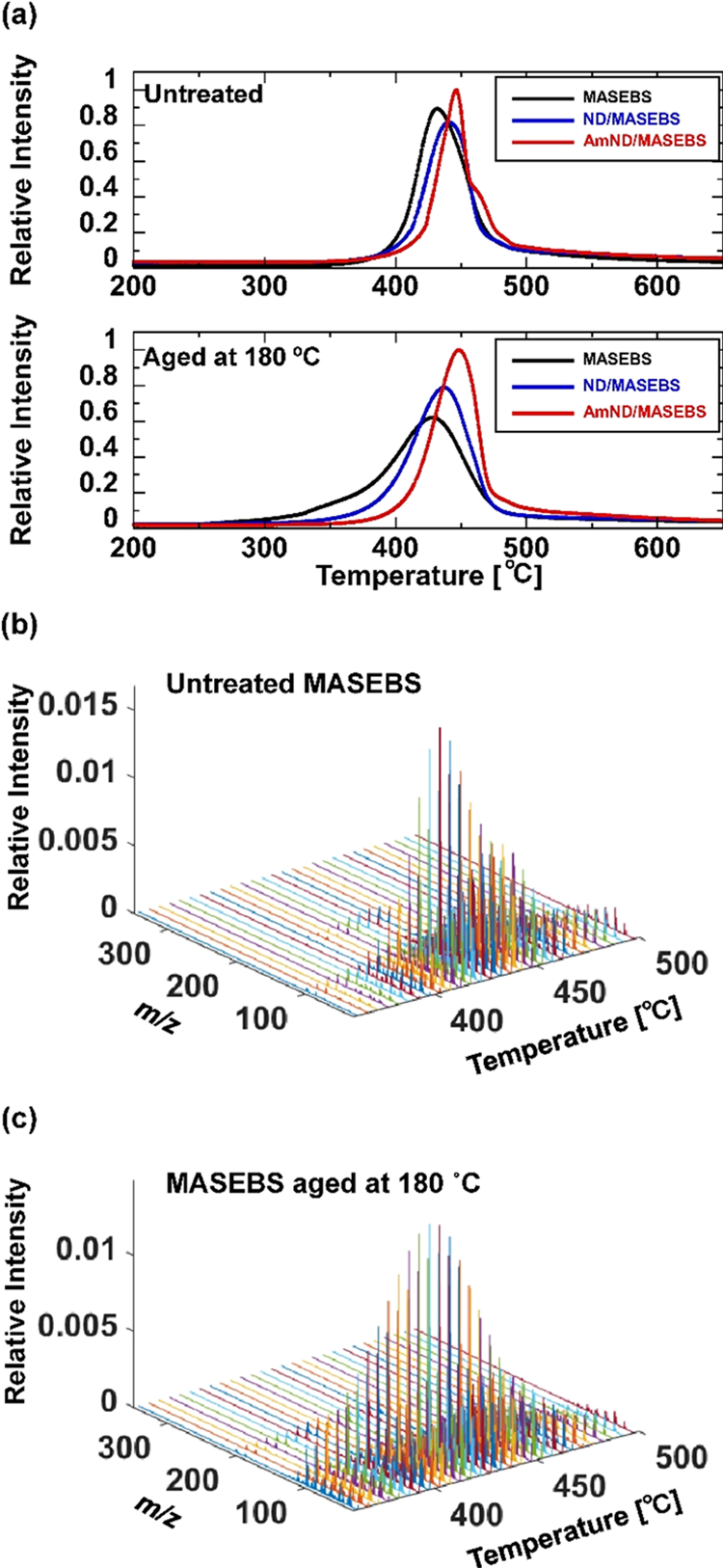
Evolution profiles of products from (a) untreated MASEBS samples and MASEBS samples aged at 180 °C observed by EGA-TOFMS in the TIC mode. ND/MASEBS and AmND/MASEBS contain 10 wt % fillers. Temperature-dependent mass spectra of the (b) untreated MASEBS and (c) MASEBS aged at 180 °C extracted during the EGA-TOFMS process.

Figure 2 shows the evolution profiles of the products from the untreated and aged MASEBS samples, which were analyzed using EGA-TOFMS in TIC mode. The aged MASEBS samples for EGA-TOFMS analyses were subjected to heating at 180 °C for 33 h under air atmosphere. This aging process caused some samples to undergo a certain degree of degradation, as confirmed by Fourier transform infrared spectrometry (Figure S4). The maximum intensities of the TIC curve for AmND/MASEBS were set as 1, and the TIC curves for the other sample were normalized by the integral values of intensity from 200 to 650 °C. Figure 2a (upper panel) shows the TIC curves of the untreated MASEBS and MASEBS composites containing 10 wt % filler. The relative intensities of the samples gradually increase and peak at around 430–450 °C before showing a decrease due to the evolution of pyrolysis products from the MASEBS component. It should be noted that the decomposition temperature showed an apparent positional shift toward a higher temperature with the inclusion of ND and AmND. This trend was more pronounced with the addition of AmNDs than with ND, suggesting that AmNDs are more effective at trapping radicals generated during the pyrolysis process in EGA.

In Figure 2a (lower panel), TIC curves of the aged MASEBS samples are displayed. Following the aging process at 180 °C, pyrolysis of the original MASEBS occurred even at lower temperatures, ranging from 225 to 425 °C, in addition to the main decomposition peak. It is suggested that the generation of unusual components with low thermal stability may be related to the progress of oxidation and scission of the polymer chains. A decrease in the decomposition temperature was also observed for ND/MASEBS after the aging treatment although the shift toward a lower temperature was smaller than that of MASEBS without any fillers. Notably, the TIC curves of AmND/MASEBS did not show a decrease in the decomposition temperature following aging, indicating that the addition of AmND effectively suppressed the formation of degradation products with low thermal stability. To understand the variations in the TIC curve throughout the aging process, the temperature-dependent mass spectra collected by EGA-TOFMS must be analyzed in detail.

Figure 2b,c illustrates the temperature-dependent mass spectra of original MASEBS, collected over the temperature region at 360–500 °C. These spectra were obtained by summing the mass spectra acquired at 5 °C intervals. Peaks arising from the decomposition products of the MASEBS constituents were observed. However, determining the intensity variations in the specific mass peaks before and after aging treatment is a laborious task. Therefore, PCA is a useful technique for elucidating subtle but pertinent information from numerous high-resolution mass spectra.

### PCA

**Figure 3:**
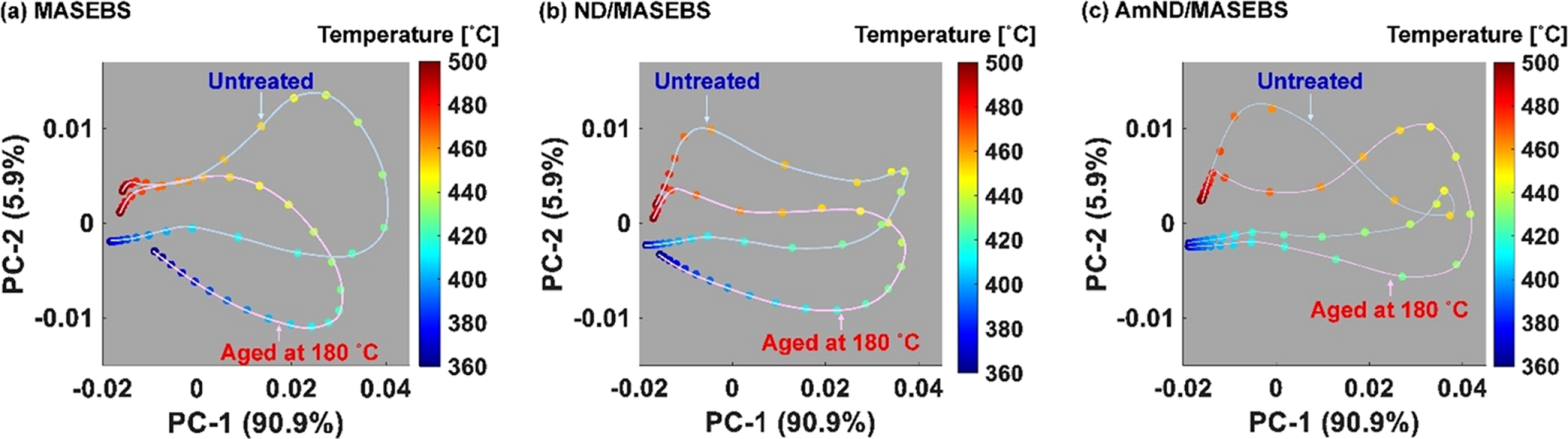
Changes in the scores of PC-1 and PC-2 derived from temperature-dependent mass spectra of untreated and aged samples collected using EGA-TOFMS; (a) MASEBS, (b) ND/MASEBS, and (c) AmND/MASEBS. The colors on the plots represent the heating temperatures during the analysis process and correspond to the color bar.

To identify specific ion series arising from decomposition of degradation products in MASEBS components, PCA was applied to the temperature-dependent mass spectra of untreated MASEBS samples and MASEBS samples aged at 180 °C for 33 h, collected by EGA-TOFMS. Figure 3 shows the score plots for PC-1 and PC-2. PC-1 and PC-2 accounted for 90.9 and 5.9% of the variance in the analyzed spectra, respectively. The score plots for MASEBS, ND/MASEBS, and AmND/MASEBS before and after aging are separately displayed in Figure 3a–c, respectively. The distance between two samples within the plot can be interpreted as the degree of dissimilarity between them. In Figure 3a, the respective sets of scores for the untreated and aged MASEBS were distributed at different positions within the two-dimensional plot, signifying that the aging treatment induced changes in the mass spectral features.

The PC-1 scores of both untreated and aged MASEBS show an apparent shift to the right in the score plot with increasing temperature, and after reaching 425 °C, they return to left side of the plot. It should be noted that the value of PC-2 of the untreated MASEBS is positioned on the upper side of the plot, while that of the aged MASEBS positioned on the lower side. In other words, the PC-2 scores can be derived from unusual decomposition products that are strongly affected by thermo-oxidative degradation. In the plots for aged MASEBS, the PC-2 score values begin to shift toward the negative side at 360 °C. Conversely, in the plots for untreated samples, the PC-2 score begin shifting toward the positive side at 440 °C. This observation suggests that the degradation products possess a lower thermal stability than pristine MASEBS.

In ND/MASEBS, the extent of the transition of PC-2 values toward the negative direction in the aged sample was limited compared to the original MASEBS (Figure 3b). Furthermore, this trend became more pronounced in AmND/MASEBS, and the PC-2 scores exhibited similar values before and after the aging process (Figure 3c). The addition of ND or AmND is presumed to contribute to the suppression of thermo-oxidative degradation in MASEBS, with particularly high suppression effects of the degradation expected from the addition of AmND.

**Figure 4:**
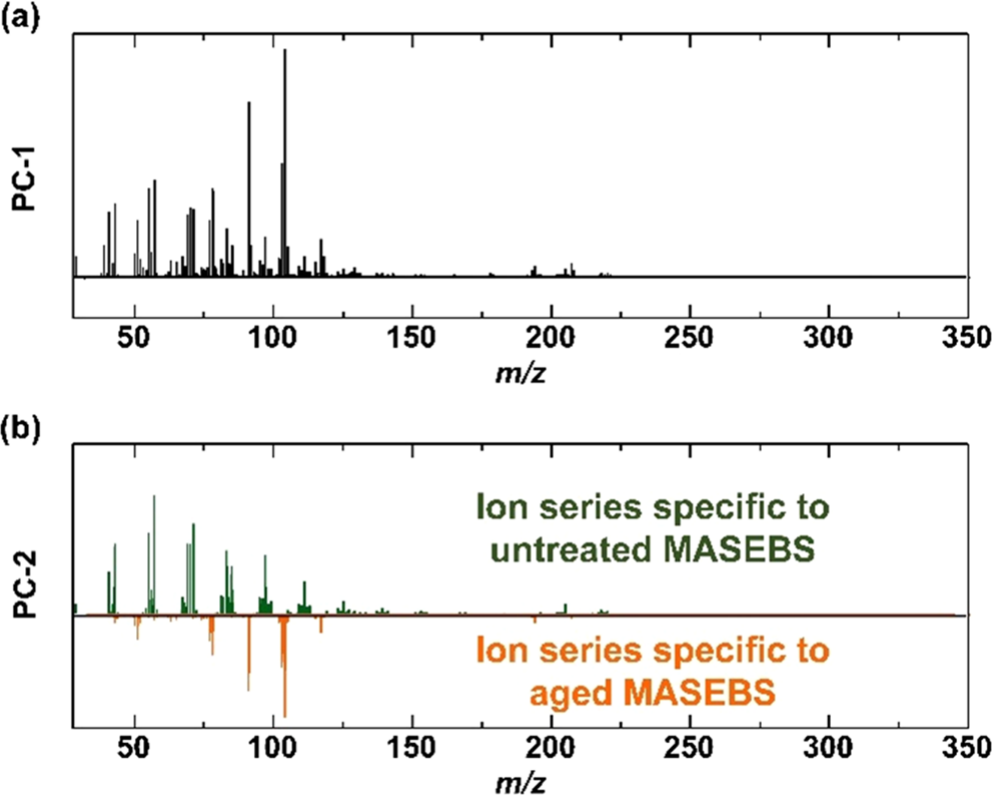
Loading plots for (a) PC-1 and (b) PC-2.

The loading plots offer a chemically or physically meaningful interpretation of the patterns observed in the score plot. Figure 4 shows the corresponding loading plots of PC-1 and PC-2, summarizing the variables in the temperature-dependent mass spectra. Figure 4a shows the loading plot of PC-1, which reveals that PC-1 is associated with an ion series that exhibits a monotonic increase or decrease depending on the pyrolysis temperature. The PC-1 score effectively captures the evolved behavior of the products for both the untreated and aged MASEBS, which agrees well with the patterns of the TIC curves in Figure 2a.

Figure 4b shows the corresponding loading plots for PC-2. The positive and negative peaks in the PC-2 loading plot contributed to the positive and negative values of the PC-2 score, respectively. From the score plot shown in Figure 3, it can be interpreted that the positive peaks in the PC-2 loading plot are linked to the ion series specific to the evolved products from the untreated MASEBS, whereas the negative peaks correspond to those originating from the aged samples. Therefore, the attribution of each peak observed during PC-2 loading appears to play a key role in understanding the complex structural changes in the MASEBS samples induced by thermo-oxidative degradation. However, these peaks were numerous because they were constructed from exact mass data, making it challenging to assign each peak comprehensively. In this context, KMD analysis was applied as an effective data mining technique for the attribution of specific ions indicated by PC-2 loading.

### KMD Analysis for Specific Ions Indicated by PC-2 Loading

**Figure 5:**
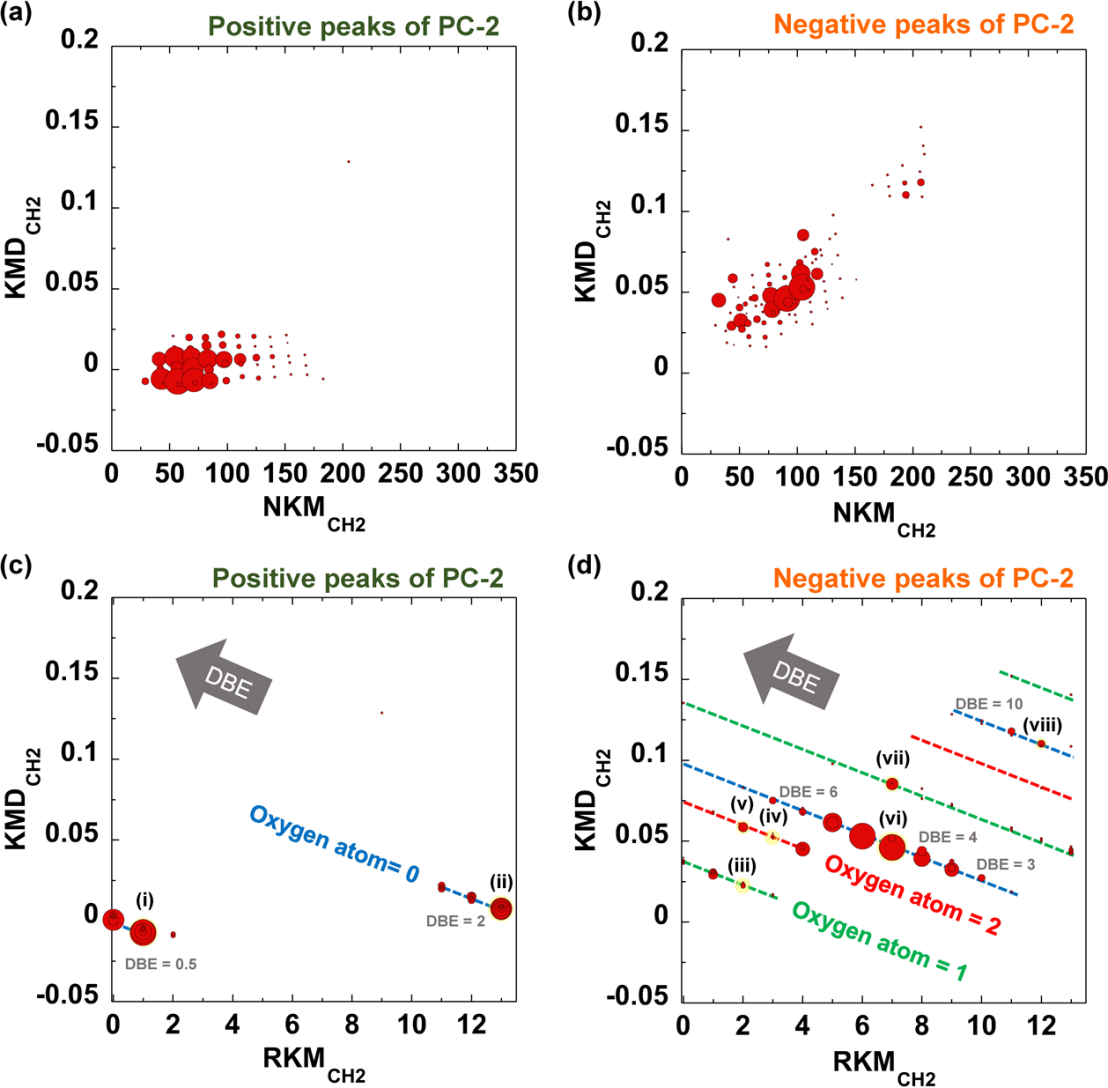
KMD and RKM plots of the PC-2 loading. KMD plots constructed from (a) positive and (b) negative peaks of PC-2 loading. RKM plots constructed from (c) positive and (d) negative peaks of PC-2 loading.

The positive and negative peaks of the PC-2 loading were transformed into KMD plots by setting CH_2_ as the base unit (Figure 5). The size of the dots in the KMD plot correlates with the number of evolved ions. In the KMD plot of the positive peaks of PC-2 loading, dots representing the distribution of hydrocarbon ions are mainly distributed in a band shape with KMD_CH_2__ = ±0.02 (Figure 5a). In contrast, the KMD plot of the negative peaks of PC-2 loading exhibits additional dots distributed at KMD_CH_2__ > 0.02, which are the ion series specific to the evolved products from aged MASEBS (Figure 5b). However, the distributions of the plots overlapped, making it difficult to distinguish individual ions clearly.

Subsequently, the KMD plots of the loading peaks for PC-2 were further converted into RKM plots, which can highlight differences in chemical structures, such as functional groups and the degree of unsaturation, by compressing the data of the distribution of carbon numbers (Figure 5c,d). In the RKM plot constructed from the positive peaks of PC-2 loading, hydrocarbon ions with different double-bond equivalents (DBE), which indicate degrees of unsaturation, were arranged diagonally, as indicated by the blue dashed line (Figure 5c). Using the molecular formula C*_c_*H*_h_*N*_n_*O*_o_*, the DBE value was calculated using eq 6.
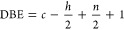
6The RKM plot revealed that the temperature-dependent mass spectra of untreated MASEBS were dominated by hydrocarbon ions (DBE 0–2.5) without any heteroatoms.

In contrast, the RKM plot derived from the negative peaks of the PC-2 loading presents two additional series (represented by green and red dashed lines) associated with ions containing one or two oxygen atoms with different DBE (Figure 5d). The formation of products derived from thermo-oxidative degradation is suggested. In addition, dots arising from highly unsaturated hydrocarbon ions with a DBE greater than 3 were observed in the RKM plot.

The representative ions chosen from ion series labeled (i–viii) in Figure 5c,d are summarized in Table 1. The attribute of each ion was determined by compositional analysis based on the exact masses. Ions (i, ii) extracted from the positive peaks of PC-2 originated from hydrocarbon ions that mainly evolved from the polyethylene (PE) or polybutylene (PB) domains in MASEBS. Ions (iii–viii) are extracted from the negative peaks of PC-2. Ions (iii, iv) were derived from ions with additional oxygen atoms compared with ion (i), which evolved from the oxidized PE and PB domains. The representative ion (v) is CO_2_^•+^, which can evolve by the decarboxylation of carbonyl compounds such as carboxylic acid and anhydride. Ion (vi) arises from monoaromatic compounds with a DBE of 4.5, such as C_7_H_7_^+^, which mainly evolves from polystyrene (PS) domain in MASEBS. Ion (vii) has one additional oxygen atom compared with ion (vi). Highly unsaturated compounds with DBE 9.0, shown as ion (viii), were mainly attributed to styrene dimers that evolved from the PS domain.

**Table 1:** Assigned Structures of Representative Peaks Observed in Loading Plot of PC-2

entry	PC-2	*m*/*z*	molecular formula	DBE	source
(i)	+	57.0697	C_4_H_9_^+^	0.5	PE or PB
	+	99.1162	C_7_H_15_^+^	0.5	PE or PB
(ii)	+	55.0548	C_4_H_7_^+^	1.5	PE or PB
	+	97.1012	C_7_H_13_^+^	1.5	PE or PB
(iii)	–	73.0665	C_4_H_9_O^+^	0.5	oxidized PE or PB
(iv)	–	101.0597	C_5_H_9_O_2_^+^	1.5	oxidized PE or PB
(v)	–	43.9912	CO_2_^•+^	2.0	oxidized MASEBS
(vi)	–	91.0571	C_7_H_7_^+^	4.5	PS
(vii)	–	105.0337	C_7_H_5_O^+^	5.5	oxidized PS
(viii)	–	208.1268	C_16_H_16_^•+^ (styrene dimer)	9.0	PS

Thus, the RKM plots enable the immediate and precise attribution of a specific ion series by the PCA output. In addition, it becomes possible to grasp the general fluctuations of ion series in the RKM plot by observing the occurrence patterns of representative ions summarized in Table 1. In the following section, the detailed evolution behavior of the representative ions is observed by the EIM mode of the EGA-TOFMS system for MASEBS, ND/MASEBS, and AmND/MASEBS to provide deeper insight into the thermo-oxidative degradation of MASEBS composites containing nanodiamonds.

### Evolution Behaviors of Representative Ions Studied in EIM Mode

**Figure 6:**
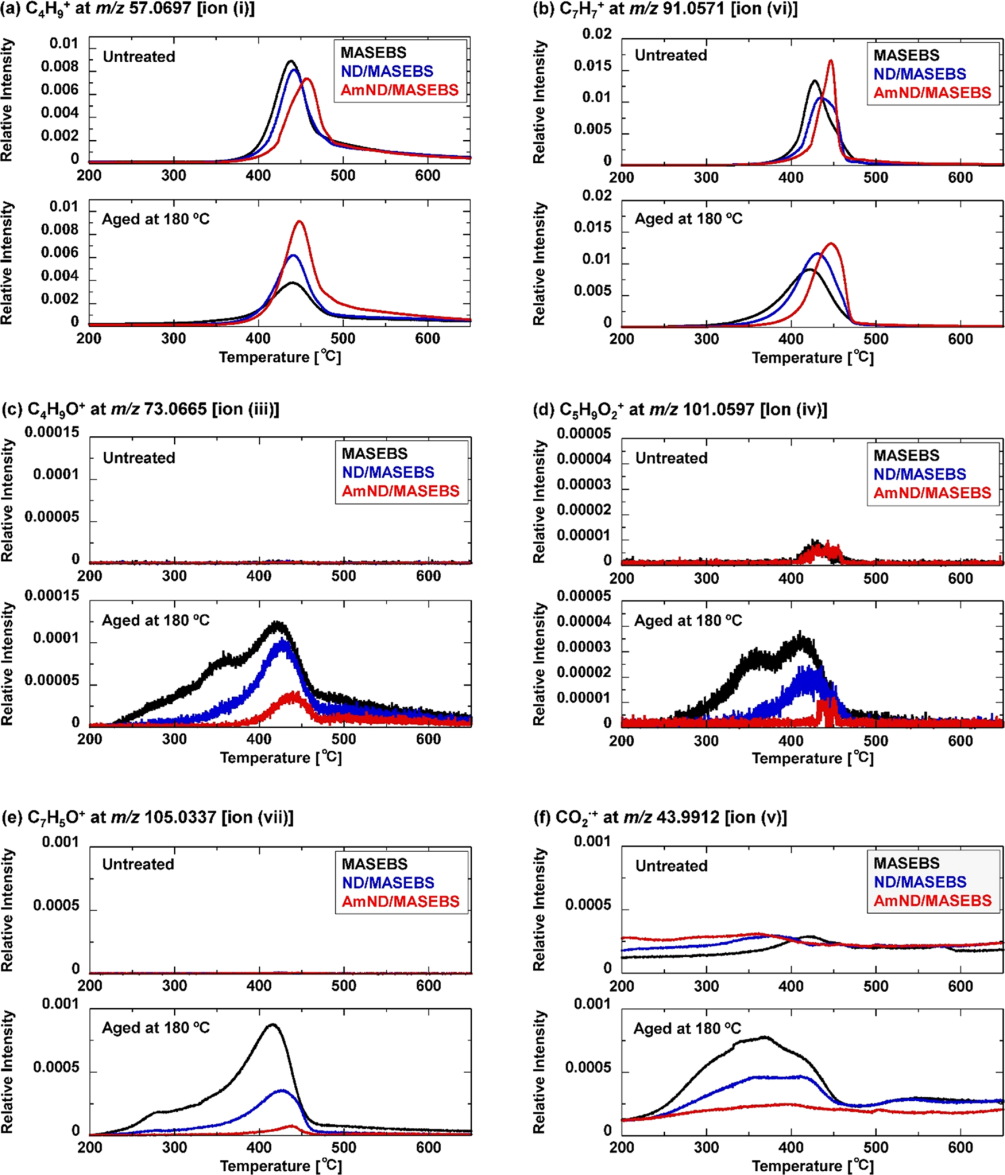
Investigation in EIM mode of the evolution behavior of specific ions to PC-2 loading attributed to (a) C_4_H_9_^+^, (b) C_7_H_7_^+^, (c) C_4_H_9_O^+^, (d) C_5_H_9_O_2_^+^, (e) C_7_H_5_O^+^, and (f) CO_2_^**•+**^.

The evolution behaviors of specific ions identified through PCA and KMD analysis were investigated for MASEBS, ND/MASEBS, and AmND/MASEBS in EIM mode (Figure 6). The evolved products of the PE and PB domains were investigated by observing the EIM curves of ion (i) at *m*/*z* 57.0697, which was attributed to C_4_H_9_^+^ (Figure 6a). Unlike the TIC curves, there was a minimal change in the evolution temperature of C_4_H_9_^+^, which was hardly observed in the EIM curves of all samples after aging treatment. However, the intensities of the EIM curves for MASEBS and ND/MASEBS significantly decreased after aging. This decline in the intensity of C_4_H_9_^+^ indicates the oxidation of the PE and PB domains and the subsequent formation of CO and CO_2_ during the aging process, which is similar to the photodegradation of polyolefins.^31^

The evolved products of the PS domain were examined by observing the EIM curves of ion (v) at *m*/*z* 91.0571, attributed to C_7_H_7_^+^ (Figure 6b). For MASEBS and ND/MASEBS, the initiation temperatures for the evolution of the C_7_H_7_^+^ ion decreased after the aging treatment, presumably due to structural changes in the PS domain through oxidation and molecular scission in a similar manner to the reported photodegradation of PS.^32^ In contrast to the evolution behavior of C_4_H_9_^+^ ions, the intensities of the C_7_H_7_^+^ ion in the EIM curves of MASEBS and ND/MASEBS were not significantly reduced. It is likely that fewer volatile compounds are generated through scission of the PS component compared to scission of the PE and PB components. Notably, the EIM curves of AmND/MASEBS showed no significant decrease in the intensity and evolution temperature of the ions after aging.

Figure 6c,d shows the evolution behavior of ion (iii) at *m*/*z* 73.0665 and ion (iv) at *m*/*z* 101.0597, attributed to C_4_H_9_O^+^ and C_5_H_9_O_2_^+^, respectively, derived from the evolved products of the oxidized PE and PB domains. No apparent peaks were observed in the EIM curves of the untreated samples (Figure 6c,d, upper panel). However, the intensities of the EIM curves of MASEBS and ND/MASEBS significantly increased after aging (Figure 6c,d, lower panel). In particular, for MASEBS, the evolution of C_4_H_9_O^+^ and C_5_H_9_O_2_^+^ occurs in a lower temperature range of 230–460 °C, indicating the formation of oxidized PE and PB domains with low thermal stability after aging. For ND/MASEBS, the increase in the intensity of the oxidized ions and the decrease in the evolution temperature were less pronounced compared with MASEBS alone. In the case of AmND/MASEBS, there was a slight increase in the intensity of C_4_H_9_O^+^ although it was limited compared with that of ND/MASEBS. The variations in the EIM curves at *m*/*z* 105.0337, attributed to the C_7_H_5_O^+^ ion, showed a similar tendency to those of C_4_H_9_O^+^ and C_5_H_9_O_2_^+^ (Figure 6e). Therefore, the PS domain was oxidized by the aging treatment, similar to the PE and PB components. Figure 6f shows the evolved behavior of ion (iv) at *m*/*z* 43.9912, attributed to CO_2_^**•+**^, which is generally similar to that of oxidized ions. It was confirmed that CO_2_^**•+**^ formation arises from the decarboxylation of the oxidized products generated during the aging process.

### Relationship between Interfacial States and Thermo-Oxidative Degradation

To explain the differences in the degradation behavior between ND/MASEBS and AmND/MASEBS, it is assumed that the key lies in the differences in the matrix–filler interfacial states. The evolutionary behavior of C_4_H_9_^+^ and C_7_H_7_^+^ in the untreated samples was examined to gain insight into the interface between the fillers and each domain in MASEBS (upper panels of Figure 6a,b). The evolution temperature of C_4_H_9_^+^ in ND/MASEBS was almost the same as that in the original MASEBS. In contrast, the evolution temperature of C_7_H_7_^+^ from ND/MASEBS shifted toward higher temperatures compared to that of the original MASEBS, possibly because of the radical-scavenging effect of ND. It is suggested that the PS domain in MASEBS molecules is adsorbed onto the ND surface via interfacial interaction, such as π–π stacking, which restricts the decomposition of the PS component in ND/MASEBS. A similar phenomenon is observed in polymer composites containing graphene.^16^ On the other hand, there can be no specific interaction between ND and PE or PB domains. By adding AmND to MASEBS, the evolution temperatures of both C_4_H_9_^+^ and C_7_H_7_^+^ shift to higher values, indicating that strong adhesion between AmND and each domain in MASEBS via the π–π stacking and chemical bonds between maleic anhydride groups in MASEBS and amino groups on the AmND surface.

The mechanical properties and filler dispersions of ND/MASEBS and AmND/MASEBS were examined using tensile testing and FE-SEM, respectively (Figures S5 and S6). The incorporation of AmNDs led to a more pronounced enhancement of the elastic modulus and stress values at 400% elongation of MASEBS than that of ND. The mechanical properties also imply the formation of rigid interfacial interactions in AmND/MASEBS.

The collective findings of this study suggest that the enhanced stability of MASEBS upon the addition of AmND for thermo-oxidative degradation can be attributed to the formation of a rigid interfacial interaction of AmND/MASEBS. Thus, it was demonstrated that EGA-PCA-KMD can obtain information on the structure and thermal stability of the degradation products.

## Conclusions

This study used EGA-PCA-KMD to investigate the structural changes in MASEBS, ND/MASEBS, and AmND/MASEBS during the thermo-oxidative degradation. Specifically, the decomposition of the original MASEBS and ND/MASEBS occurred at lower temperatures after aging, whereas the TIC curves of AmND/MASEBS showed no significant change in the pyrolysis temperature. This study primarily aimed to probe the fine details within the temperature-dependent mass spectra collected using EGA-TOFMS to provide a meaningful interpretation of the changes in the TIC curve throughout the aging process. To this end, PCA was performed on the temperature-dependent mass spectra of both untreated and aged MASEBS to identify the specific ion series that evolved from the degradation products in MASEBS. The variation in the PC-2 scores correlated with the pyrolysis behavior associated with changes in the mass spectra due to MASEBS degradation. The mass peaks extracted through PC-2 loading were further attributed to KMD analysis. The mass spectra of the untreated MASEBS were enriched in hydrocarbon ions originating from the PE and PB domains, whereas the aged MASEBS exhibited a higher abundance of ions containing oxygen atoms and aromatic ions that evolved from the oxidized MASEBS and PS domains, respectively.

The detailed evolutionary behaviors of the representative ions were observed using the EIM mode of the EGA-TOFMS system in MASEBS, ND/MASEBS, and AmND/MASEBS. The evolution of pyrolysis products from oxidized MASEBS molecules was significantly suppressed in AmND/MASEBS compared to ND/MASEBS. According to the evolution behavior of the pyrolysis products from each domain in MASEBS molecules, the addition of AmND contributed to improvement in the stability toward thermo-oxidative degradation by the formation of rigid interfacial interaction between AmND and each domains in MASEBS via the π–π stacking and chemical bonds.

The novel integrated approach EGA-PCA-KMD proposed in this study offers a comprehensive understanding of the degradation mechanism by obtaining information on the detailed structural changes of the thermo-oxidative degradation of polymer materials.

## Supporting Information Available

Schematic of the interfacial structure of AmND/MASEBS (Figure S1), FE-SEM images of the nanodiamonds (Figure S2), Raman spectra of the nanodiamonds (Figure S3), isothermal in situ FTIR measurements (Figure S4), mechanical properties of the MASEBS samples (Figure S5), FE-SEM images of the MASEBS nanocomposites. (Figure S6), difference spectrum (Figure S7), verification of reproducibility in EGA-TOFMS measurements (Figure S9) (PDF)

## References

[ref1] Pfaendner R. How Will Additives Shape the Future of Plastics?. Polym. Degrad. Stab. 2006, 91, 2249–2256. 10.1016/j.polymdegradstab.2005.10.017.

[ref2] Dintcheva N. T.; D’Anna F. Anti-/Pro-oxidant Behavior of Naturally Occurring Molecules in Polymers and Biopolymers: A Brief Review. ACS Sustainable Chem. Eng. 2019, 7, 12656–12670. 10.1021/acssuschemeng.9b02127.

[ref3] Ojeda T.; Freitas A.; Birck K.; Dalmolin E.; Jacques R.; Bento F.; Camargo F. Degradability of Linear Polyolefins Under Natural Weathering. Polym. Degrad. Stab. 2011, 96, 703–707. 10.1016/j.polymdegradstab.2010.12.004.

[ref4] Ata S.; Tomonoh S.; Yamda T.; Hata K. Improvement in Thermal Durability of Fluorinated Rubber by the Addition of Single-Walled Carbon Nanotubes as a Thermally Stable Radical Scavenger. Polymer 2017, 119, 112–117. 10.1016/j.polymer.2017.05.025.

[ref5] Ata S.; Hayashi Y.; Nguyen Thi T. B. N.; Tomonoh S.; Kawauchi S.; Yamada T.; Hata K. Improving Thermal Durability and Mechanical Properties of Poly(Ether Ether Ketone) with Single-Walled Carbon Nanotubes. Polymer 2019, 176, 60–65. 10.1016/j.polymer.2019.05.028.

[ref6] Xu X.; You Y.; Liu X.; Wei D.; Guan Y.; Zheng A. Experimental and Density Functional Theory Investigations on the Antioxidant Mechanism of Carbon Nanotubes. Carbon 2021, 177, 189–198. 10.1016/j.carbon.2021.02.077.

[ref7] Watts P. C. P.; Fearon P. K.; Hsu W. K.; Billingham N. C.; Kroto H. W.; Walton D. R. M. Carbon Nanotubes as Polymer Antioxidants. J. Mater. Chem. 2003, 13, 491–495. 10.1039/b211328g.

[ref8] Morlat-Therias S.; Fanton E.; Gardette J. L.; Peeterbroeck S.; Alexandre M.; Dubois P. Polymer/Carbon Nanotube Nanocomposites: Influence of Carbon Nanotubes on EVA Photodegradation. Polym. Degrad. Stab. 2007, 92, 1873–1882. 10.1016/j.polymdegradstab.2007.06.021.

[ref9] Tang M.; Xing W.; Wu J.; Huang G.; Xiang K.; Guo L.; Li G. Graphene as a Prominent Antioxidant for Diolefin Elastomers. J. Mater. Chem. A 2015, 3, 5942–5948. 10.1039/C4TA06991A.

[ref10] Prosheva M.; Aboudzadeh M. A.; Leal G. P.; Gilev J. B. High-Performance UV Protective Waterborne Polymer Coatings Based on Hybrid Graphene/Carbon Nanotube Radicals Scavenging Filler. Part. Part. Syst. Charct. 2019, 36, 180555 10.1002/ppsc.201800555.

[ref11] Tsuge S.; Ohtani H.; Watanabe C. Pyrolysis-GC/MS Data Book of Synthetic Polymers; Elsevier BV: Oxford, UK, 2011.

[ref12] Sato H.; Tsuge S.; Ohtani H.; Aoi K.; Takasu A.; Okada M. Characterization of Chitin-Based Polymer Hybrids by Temperature-Programmed Analytical Pyrolysis Techniques. 1. Chitin-graft-poly(2-methyl-2oxazoline)/poly(vinyl chloride) blends. Macromolecules 1997, 30, 4030–4037. 10.1021/ma9702399.

[ref13] Sato H.; Ohtani H.; Tsuge S.; Aoi K.; Takasu A.; Okada M. Characterization of Chitin-Based Polymer Hybrids by Temperature-Programmed Analytical Pyrolysis Techniques. 2. Chitin-graft-poly(2-methyl-2-oxazoline)/poly(vinyl alcohol) brends. Macromolecules 2000, 33, 357–362. 10.1021/ma991123a.

[ref14] Sato H.; Ohtani H.; Harada R.; Tsuge S.; Kato M.; Usuki A. Polymer/Silicate Interaction in Nylon 6-Clay Hybrid Studied by Temperature Programmed Pyrolysis Techniques. Polym. J. 2006, 38, 171–177. 10.1295/polymj.38.171.

[ref15] Watanabe R.; Hagihara H.; Sato H. Structure–Property Relationships of Polypropylene-Based Nanocomposites Obtained by Dispersing Mesoporous Silica into Hydroxyl-Functionalized Polypropylene. Part 2: Matrix–Filler Interactions and Pore Filling of Mesoporous Silica Characterized by Evolved Gas Analysis. Polym. J. 2018, 50, 1067–1077. 10.1038/s41428-018-0096-9.

[ref16] Watanabe R.; Sugahara A.; Hagihara H.; Mizukado J.; Shinzawa H. Three-Way Evolved Gas Analysis-Mass Spectrometry Combined with Principal Component Analysis (EGA-MS-PCA) to Probe Interfacial States between Matrix and Filler in Poly(Styrene-b-Butadiene-b-Styrene) (SBS) Nanocomposites. Polym. Test. 2021, 101, 107300 10.1016/j.polymertesting.2021.107300.

[ref17] Ishida T.; Kitagaki R.; Elakneswaran Y.; Mizukado J.; Shinzawa H.; Sato H.; Hagihara H.; Watanabe R. Network Degradation Assessed by Evolved Gas Analysis–Mass Spectrometry Combined with Principal Component Analysis (EGA–MS–PCA): A Case of Thermo-Oxidized Epoxy/Amine Network. Macromolecules 2023, 56, 883–891. 10.1021/acs.macromol.2c02383.

[ref18] Næs T.; Isaksson T.; Fearn T.; Davies T. A User-Friendly Guide to Multivariate Calibration and Classification; NIR Publications Publications: Chichester, 2002.

[ref19] Vandeginste B. G. M.; Massart D. L.; Buydens L. M. C.; De Jong S.; Lewi P. J.; Smeyers-Verbeke J. Handbook of Chemometrics and Qualimetrics; Elsevier: Amsterdam, 1998.

[ref20] Wold S.; Esbensen K.; Geladi P. Principal Component Analysis. Chemom. Intell. Lab. Syst. 1987, 2, 37–52. 10.1016/0169-7439(87)80084-9.

[ref21] Beebe K. R.; Pell R. J.; Seasholtz M. B. Chemometrics: A Practical Guide; John Wiley & Sons: New York, 1998.

[ref22] Watanabe R.; Oishi A.; Nakamura S.; Hagihara H.; Shinzawa H. Real-Time Monitoring of the Thermooxidative Degradation Behavior of Poly(Acrylonitrile-Butadiene-Styrene) Using Isothermal In-Situ Fourier Transform Infrared Spectroscopy Combined With Principal Component Analysis. Polymer 2023, 283, 126243 10.1016/j.polymer.2023.126243.

[ref23] Hughey C. A.; Hendrickson C. L.; Rodgers R. P.; Marshall A. G.; Qian K. Kendrick Mass Defect Spectrum: A Compact Visual Analysis for Ultrahigh-Resolution Broadband Mass Spectra. Anal. Chem. 2001, 73, 4676–4681. 10.1021/ac010560w. 11605846

[ref24] Nakamura S.; Watanabe R.; Yamane S.; Sato H. Kendrick Mass Defect Analysis-Based Data Mining Technique for Trace Components in Polyolefins Observed by Pyrolysis-Gas Chromatography/High-Resolution Mass Spectrometry. J. Anal. Appl. Pyrol. 2023, 170, 105910 10.1016/j.jaap.2023.105910.

[ref25] Sato H.; Nakamura S.; Teramoto K.; Sato T. Structural Characterization of Polymers by MALDI Spiral-TOF Mass Spectrometry Combined With Kendrick Mass Defect Analysis. J. Am. Soc. Mass Spectrom. 2014, 25, 1346–1355. 10.1007/s13361-014-0915-y. 24845357 PMC4105590

[ref26] Yamane S.; Fouquet T. N. J.; Nakamura S.; Sato H.; Mizukado J. A Data Mining Method From Pyrolyzed Products: Pyrolysis-Gas Chromatography-Photoionization-High Resolution Time-Of-Flight Mass Spectrometry and Kendrick Mass Defect Analysis for Polymer Semiconductor Poly(3-Hexylthiophene). J. Anal. Appl. Pyrol. 2020, 151, 104923 10.1016/j.jaap.2020.104923.

[ref27] Nakamura S.; Cody R. B.; Sato H.; Fouquet T. Graphical Ranking of Divisors to Get the Most out of a Resolution-Enhanced Kendrick Mass Defect Plot. Anal. Chem. 2019, 91, 2004–2012. 10.1021/acs.analchem.8b04371. 30582791

[ref28] Fouquet T.; Satoh T.; Sato H. First Gut Instincts Are Always Right: The Resolution Required for a Mass Defect Analysis of Polymer Ions Can Be as Low as Oligomeric. Anal. Chem. 2018, 90, 2404–2408. 10.1021/acs.analchem.7b04518. 29336551

[ref29] Fouquet T.; Sato H. Extension of the Kendrick Mass Defect Analysis of Homopolymers to Low Resolution and High Mass Range Mass Spectra Using Fractional Base Units. Anal. Chem. 2017, 89, 2682–2686. 10.1021/acs.analchem.6b05136. 28194938

[ref30] Watanabe R.; Sugahara A.; Hagihara H.; Mizukado J.; Shinzawa H. In Situ Fourier Transform Infrared Spectroscopic Imaging for Elucidating Variations in Chemical Structures of Polymer Composites at the Matrix-Filler Interface During Reactive Processing. Macromolecules 2020, 53, 10711–10717. 10.1021/acs.macromol.0c01878.

[ref31] Gijsman P.; Meijers G.; Vitarelli G. Comparison of the UV-Degradation Chemistry of Polypropylene, Polyethylene, Polyamide 6 and Polybutylene Terephthalate. Polym. Degrad. Stab. 1999, 65, 433–441. 10.1016/S0141-3910(99)00033-6.

[ref32] Liu X.; Hu K. Studies on Photoprotection and Photo-oxidation in Polystyrene With Various Wavelength Regions. Polym. Adv. Technol. 1996, 7, 117–121. 10.1002/(SICI)1099-1581(199602)7:2<117::AID-PAT454>3.0.CO;2-N.

