# Supplementary material for: Revealing Molecular-Scale Structural Changes in Polymer Nanocomposites during Thermo-Oxidative Degradation Using Evolved Gas Analysis with High-Resolution Time-of-Flight Mass Spectrometry Combined with Principal Component Analysis and Kendrick Mass Defect Analysis
Source: Anal Chem. 2024 Jan 30;96(6):2628–36. doi: 10.1021/acs.analchem.3c05269 (PMC10867796; doi:10.1021/acs.analchem.3c05269)
Supplement: Supplementary file 1 — ac3c05269_si_001.pdf [file ac3c05269_si_001.pdf]

Supporting information

Revealing molecular-scale structural changes in polymer nanocomposites during thermo-oxidative degradation using evolved gas analysis with high-resolution time-of-flight mass spectrometry combined with principal component analysis and Kendrick mass defect analysis

Ryota Watanabe[a*], Sayaka Nakamura[a], Aki Sugahara[a], Mayumi Kishi[a], Hiroaki Sato[a], Hideaki Hagihara[a], and Hideyuki Shinzawa[a]

[a]Research Institute for Sustainable Chemistry, National Institute of Advanced Industrial Science and Technology (AIST), 1-1-1 Higashi, Tsukuba 305-8565, Japan

Tel: +81-29-861-3709, Fax: +81-29-861-4457

Correspondence authors.

E-mail address: r.watanabe@aist.go.jp (R. Watanabe)

TABLE OF CONTENTS

1. Schematic of interfacial structure of AmND/MASEBS ········ S3

2. Morphology of nanodiamonds ········ S3

3. Carbon component of ND and AmND ········ S4

4. Isothermal *in-situ* Fourier transform infrared (FTIR) spectrometry ········ S5

5. Mechanical properties and filler dispersion state ········ S7

6. Analysis of difference spectrum ········ S11

7. Verification of the reproducibility of EGA-MS measurements ········ S14

1. Schematic of interfacial structure of AmND/MASEBS

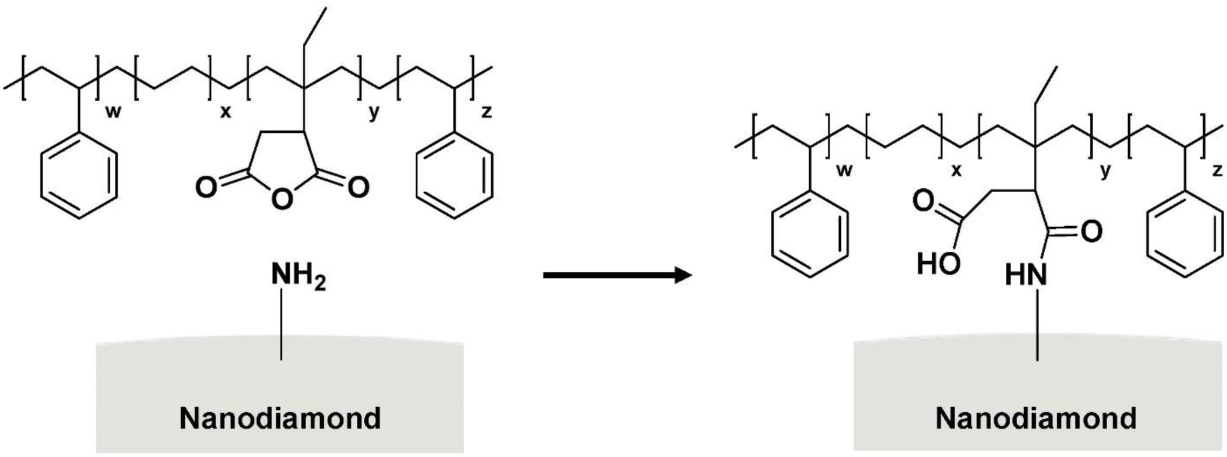

Figure S1. Schematic of the structural change at the interface of AmND/MASEBS.

2. Morphology of nanodiamonds

The morphology of nanodiamonds was evaluated using Field-emission scanning electron microscopy (FE-SEM, S-4800, Hitachi High-Tech Science Corporation, Japan) operated at 1 kV in secondary electron mode.

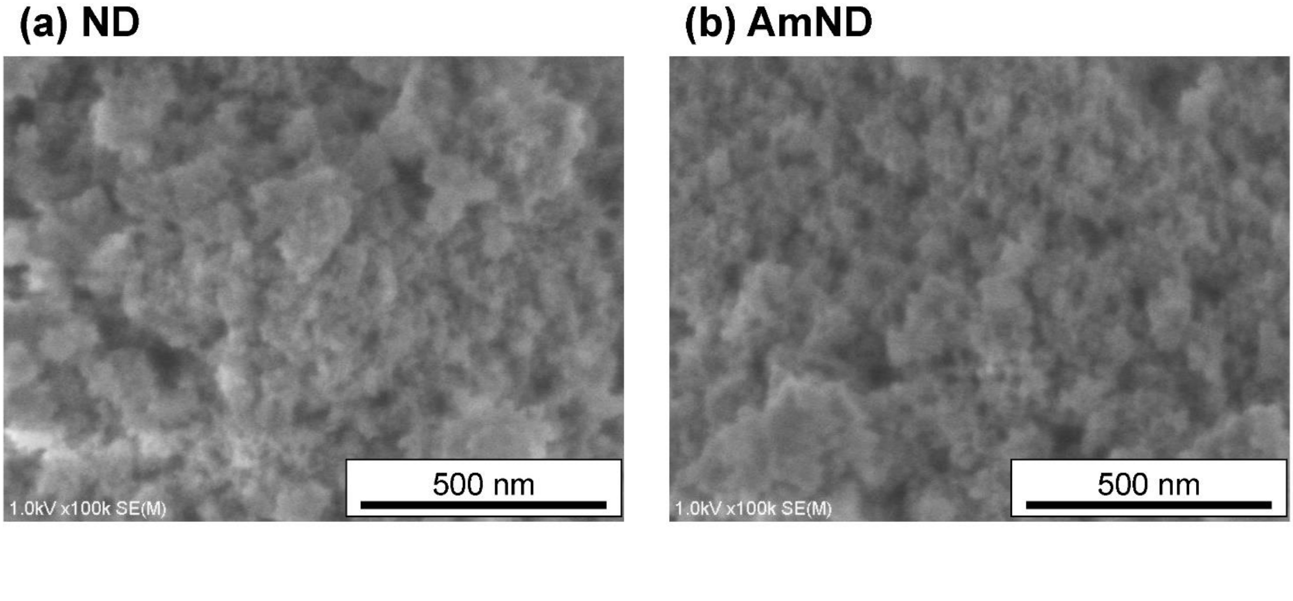

Figure S2. FE-SEM images of (a) ND and (b) AmND.

3. Carbon component of ND and AmND

Raman spectra of the ND and AmND were recorded using a Raman spectrometer (NRS-4500, Jasco corporation, Japan) equipped with a 532 nm argon-ion laser. All the Raman spectra were recorded at room temperature with a resolution of 4.8 cm^{-1}. The peak at near 1340 cm^{-1} is identified as defect induced disorder mode (D band), which generally arises from sp^3 carbon samples. The other prominent peak observed at near 1580 cm^{-1} is identified as the tangential mode (G band) caused by the in-plane vibrational motion of the sp^2 carbon. Ratios of the intensities of D and G band (I_D/I_G) of ND and AmND are 1.517 and 0.782, indicating that a higher content of graphitic carbon in the AmND compared to that in the ND (Figure S3). In the case of carbon nanotubes, radical scavenging efficiency is commonly increased with increasing I_D/I_G ratio.[1,2]

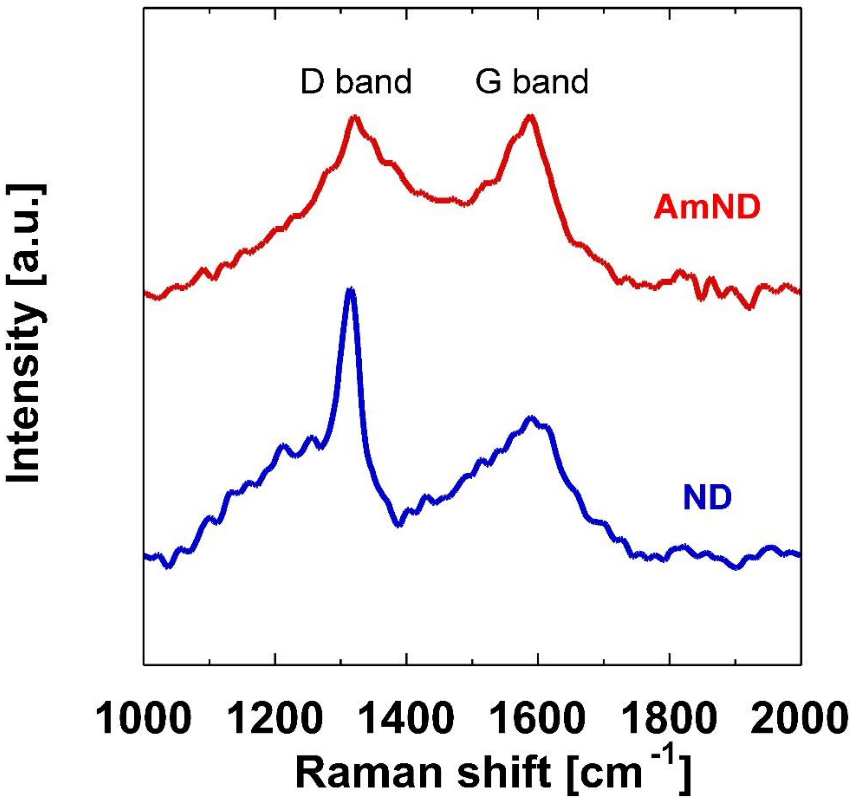

Figure S3. Raman spectra of ND and AmND.

4. Isothermal *in-situ* Fourier transform infrared (FTIR) spectrometry

ND/MASEBS and AmND/MASEBS were fabricated by mixing 30 wt% of fillers in 15 mL of toluene solution containing 2.7 g of MASEBS at room temperature using magnetic stirring. Dried composite samples were obtained by evaporation of toluene solvent overnight at room temperature. The thin films of MASEBS samples with a thickness of 50 μm for FTIR analysis were prepared by hot pressing at 200 ˚C. *In-situ* FTIR measurements were performed according to the method described previously[3] using an FTIR spectrometer (Nicolet 6700, Thermo Scientific, USA) coupled with a GladiATR (PIKE Technologies, USA) equipped with a diamond attenuated total reflection (ATR)

prism for monitoring the degradation state. The FTIR spectra were averaged over 256 scans at a resolution of 4 cm^{-1}. were placed on an ATR prism heated to 180 °C under air. The interval between spectral collections was 10 min. The data were acquired using the TempPRO 7 software (PIKE Technologies, USA) and subsequently imported into MATLAB version R2022b (MathWorks, USA). The spectra were pretreated with Savitzky–Golay 11 point-smoothing and subjected to a linear offset baseline correction.

Isothermal *in-situ* FTIR analysis at 180 °C under air atmosphere was performed to roughly understand the thermooxidative stability of the MASEBS samples (Figure S4). Figure S4a–c shows the time-dependent FTIR spectra of MASEBS, ND/MASEBS and AmND/MASEBS in the 1830–1400 cm^{-1} region. The absorption intensities of C=O groups at around 1750–1650 cm^{-1} arising from ketones, aldehydes and carboxylic acids[4] generated by oxidation of MASEBS molecules tend to increase with prolonging heating time at 180 °C. Variations in the intensity ratios of C=O band at 1734 cm^{-1} to band at 1491 cm^{-1} attributed to scissoring mode of CH_2 groups of polystyrene (I_{1734}/I_{1491}) are estimated from the time-dependent FTIR spectra to evaluate the formation behavior of the C=O groups during aging process (Figure S4d). In the case of the original MASEBS, as the aging time increases from 0 to 10 h, I_{1734}/I_{1491} increases more rapidly and forms a plateau. By increasing in the content of ND or AmND in MASEBS, the rise in I_{1734}/I_{1491} is mitigated. Particularly, the addition of AmND had more significant effect in reducing the increase in the intensity ratio compared to that of ND. MASEBS containing 10 wt% of AmND does not show a substantial increase in I_{1734}/I_{1491} during the aging process. Although

FTIR measurements provide information on the oxidation progression of MASEBS matrix by probing C=O groups, it is difficult to study the detailed structure and thermal stability of degradation products for better understanding of the radical trapping efficiency of nanodiamond addition. Hence, it can be an important task to further investigate Thermooxidative degradation behaviors of the polymer samples by EGA-TOFMS system.

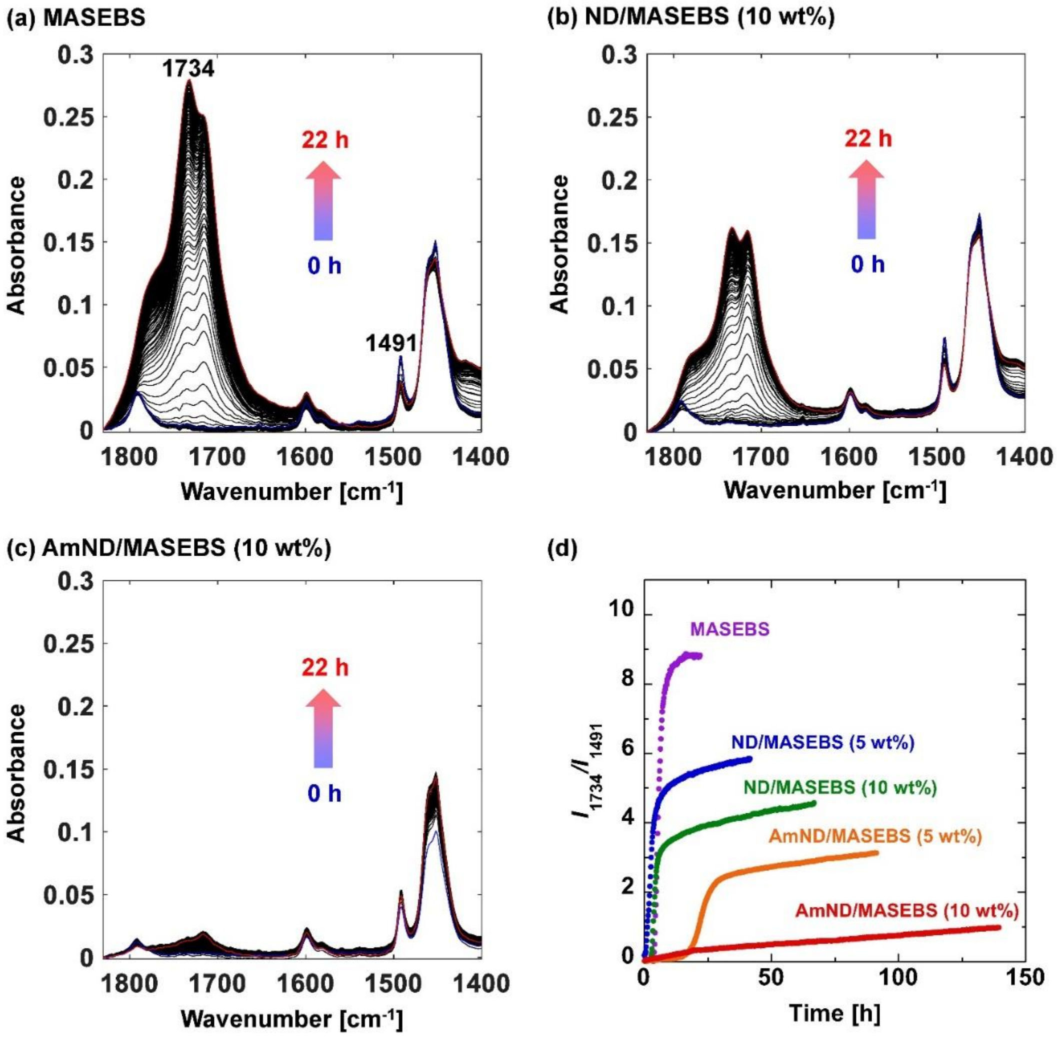

Figure S4. Time-dependent FTIR spectra of (a) MASEBS and (b) ND/MASEBS and (c) AmND/MASEBS containing 10 wt% of fillers. (d) Variations in the intensity ratios of 1734 cm^{-1} / 1491 cm^{-1} (I_{1734}/I_{1491}) estimated from the time-dependent FTIR spectra of MASEBS samples.

5. Mechanical properties and filler dispersion state

Sample sheets (50 × 50 × 0.5 mm) for tensile testing were prepared using 2 g of the dried composites by hot pressing at 200 °C under 5 MPa for 3 min, and then under 10 MPa for 10 min, using a Naflon® sheet (Nichias), a stainless steel window frame 0.5 mm in thickness and stainless steel plates. The hot-pressed samples were then quickly quenched to room temperature. Tensile tests were conducted using a multi-purpose stretching tester (EZ-LX, Shimadzu, Japan) using a dumbbell-shaped specimen (0.5 mm thick, 4 mm wide and 15 mm long in the parallel part) at a crosshead speed of 10 mm/min at 25 °C.

Three specimens were tested for each sample. The mechanical properties of MASEBS, ND/MASEBS and AmND/MASEBS were examined by tensile testing (Figure S5). The representative stress-strain curves of MASEBS and MASEBS nanocomposites containing 10 wt% of fillers are shown in Figure S5a. Filler addition and surface modification of ND with amino groups tend to enhance stress values during tensile testing without any significant changes in ductility. Mechanical properties calculated from the stress-strain curves are summarized in Figure S5b,c. The values of elastic modulus and stress at 400% strain reveal that the properties of MA-SEBS are enhanced by the addition of fillers. Particularly, the addition of AmND resulted in a more efficient increase in the elastic modulus and stress values at 400% of MASEBS compared to that of ND.

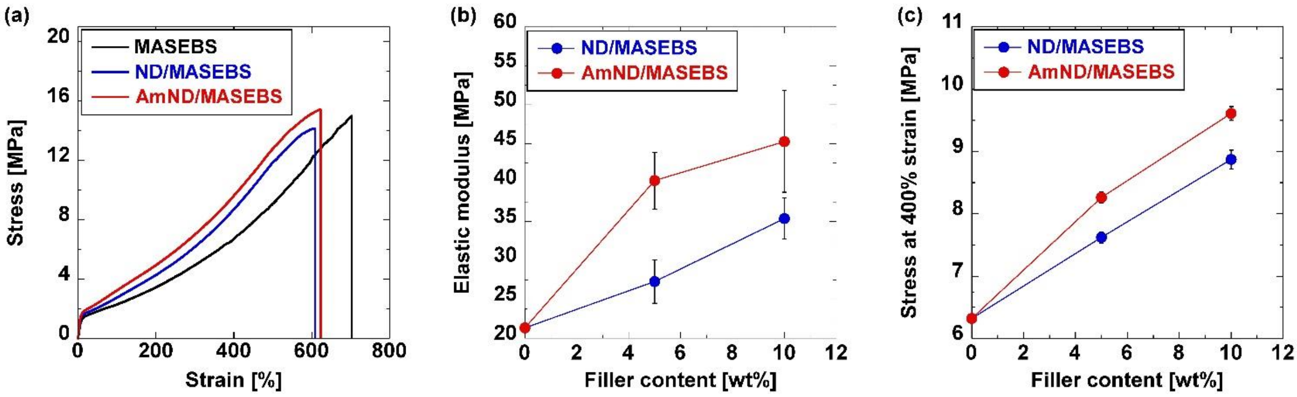

Figure S5. Mechanical properties of the original MASEBS, ND/MASEBS and AmND/MASEBS; (a) stress-strain curves of the original MASEBS and nanocomposites containing 10 wt% of fillers, (b) elastic modulus, and (c) stress at 400% strain.

The FE-SEM images of freeze-fracture surfaces of the ND/MASEBS and AmND/SEBS sheets are displayed in Figure S6. It was demonstrated that both ND and AmND are homogeneously dispersed in MASEBS at nanoscale and their dispersibility are almost unchanged. Therefore, the difference in the mechanical properties between ND/MASEBS and AmND/MASEBS appears to derive from variation in matrix-filler interfacial adhesion. It is likely that the formation of a larger number of interfacial covalent bonds between matrix and filler (Figure S1) contribute to mobility restriction of MA-SEBS around the interface, resulting in the improved strength observed in AmND/MASEBS.

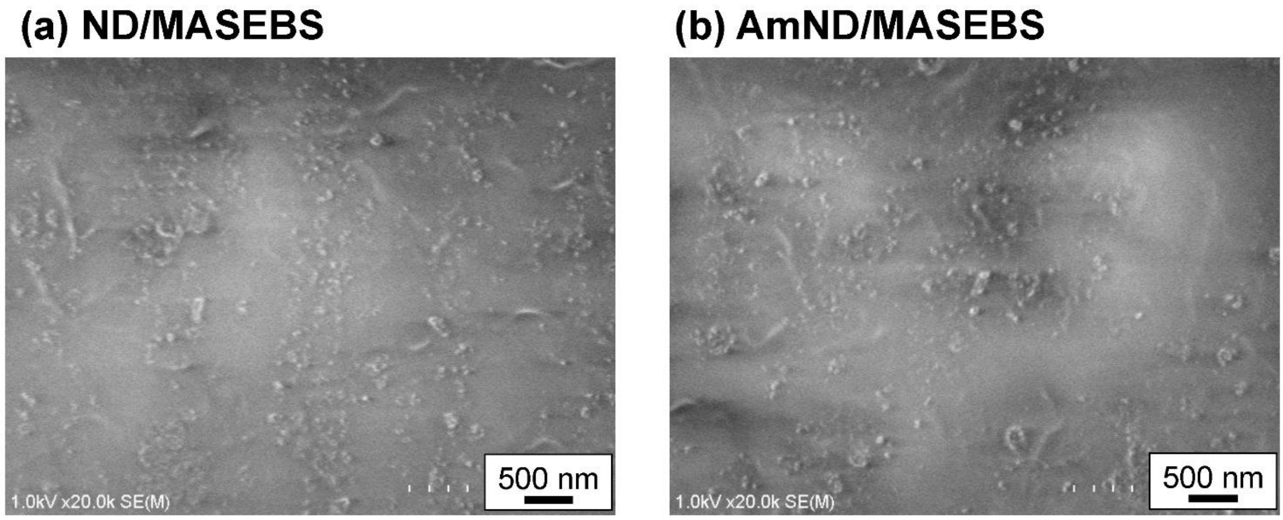

Figure S6. FE-SEM images of (a) ND/MASEBS and (b) AmND/MASEBS. The granular white contrast areas in the FE-SEM images indicate the fillers.

6. Analysis of difference spectrum

The difference spectrum was generated from two average mass spectra calculated from temperature-dependent mass spectra measured for untreated and aged MASEBS (Figure S7). Average mass spectra of untreated and aged MASEBS are shown in Figure S7a and b, respectively. Figure S7c represents the difference spectrum created by subtracting the mass spectrum of aged MASEBS from that of untreated MASEBS. The negative components in the difference spectrum are predominantly present in the aged MASEBS.

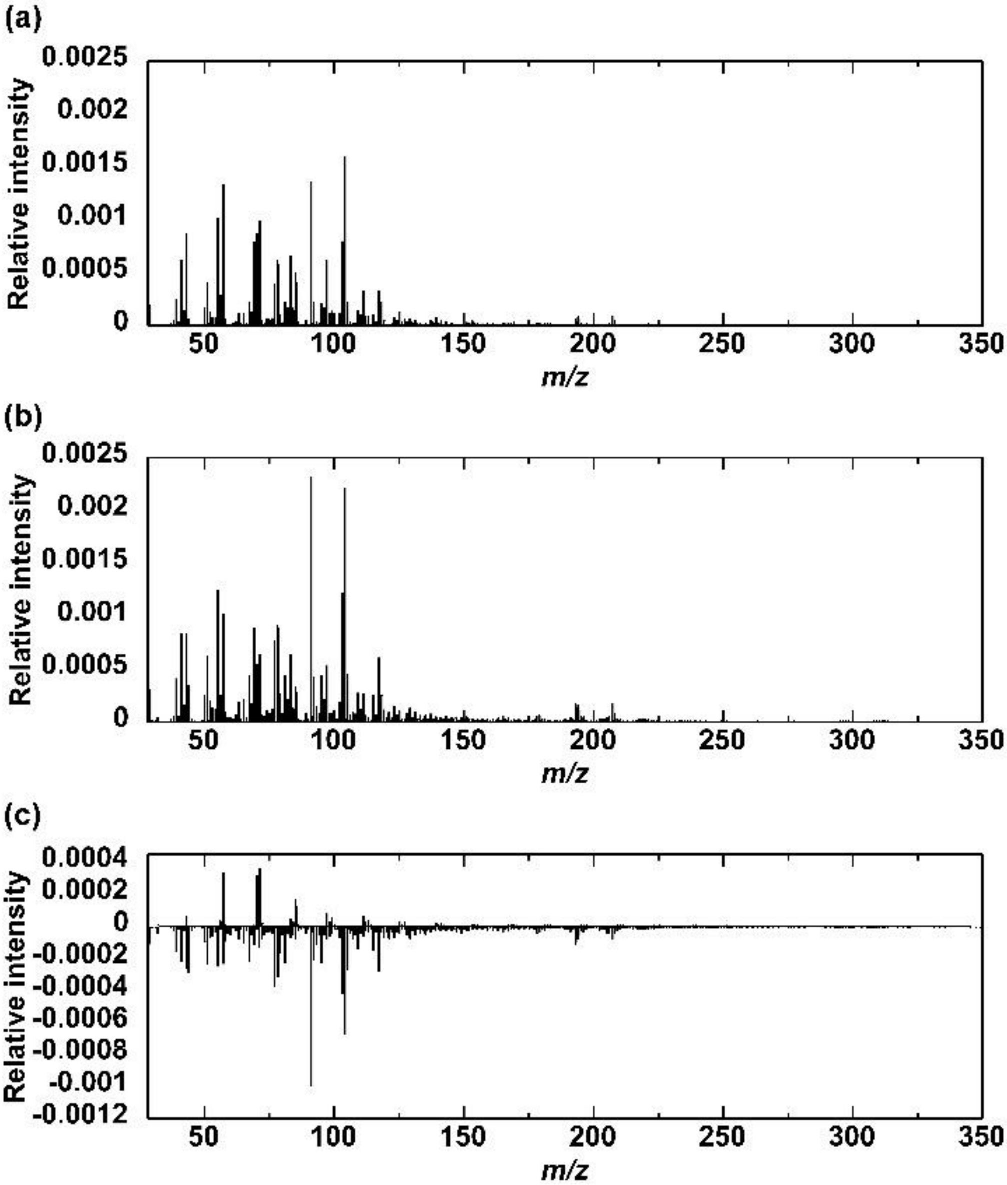

Figure S7. Difference spectrum generated from two average mass spectra calculated from temperature-dependent mass spectra measured for untreated and aged MASEBS collected by EGA-TOFMS over the temperature region at 200–650 °C.

The positive and negative peaks of the difference spectrum were transformed into KMD plots by setting CH_2 as the base unit (Figure S8). In the KMD plot of the positive peaks of difference spectrum,

dots representing the distribution of hydrocarbon ions are mainly distributed in a band shape with $KMD_{CH2} = \pm 0.02$ (Figure S8a). In contrast, the KMD plot of the negative peaks of difference spectrum exhibits additional dots distributed at $KMD_{CH2} > 0.02$, which are the ion series specific to the pyrolysis products evolved from aged MASEBS (Figure S8b). However, the distributions of the plots overlapped, making it difficult to distinguish individual ions clearly. The KMD plots of the peaks for difference spectrum were further converted into RKM plots, which can highlight differences in chemical structures, such as functional groups and the degree of unsaturation, by compressing the data of the distribution of carbon numbers (Figure S8c and d). The RKM plot shown in Figure S8c and d was different with Figure 5c and d in the main text. For example, there is an overlap in the distribution of hydrocarbon ions located at $KMD_{CH2} = \pm 0.02$ that mainly evolved from the polyethylene (PE) or polybutylene (PB) domains in MASEBS. This is mainly because the difference spectrum directly captures the variability in the shape of mass spectra for each measurement. The information obtained with PC-2 in this study takes advantage of the excellent characteristics of PCA to selectively extract spectral components related to thermos-oxidative degradation products from MASEBS.

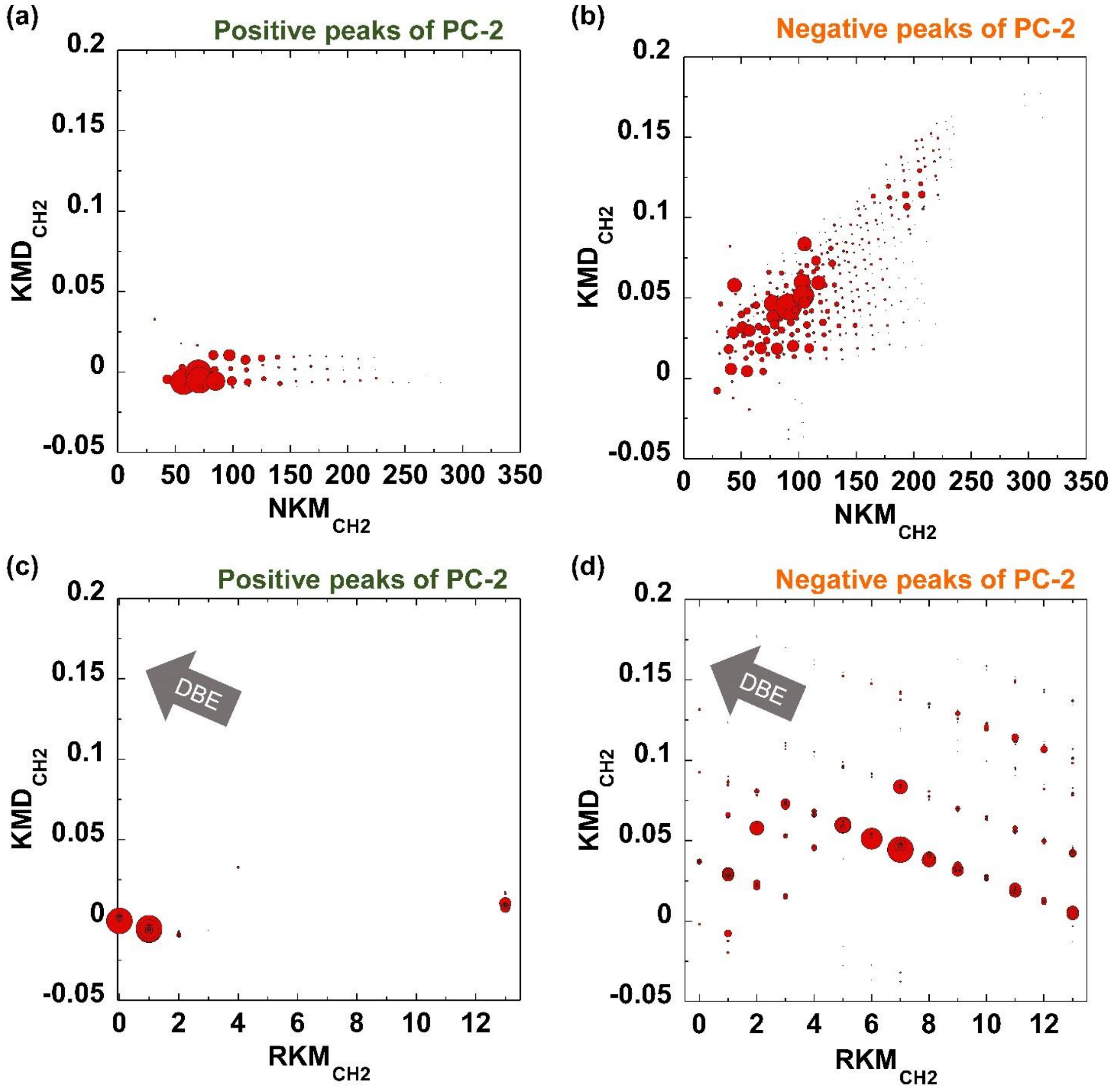

Figure S8. KMD and RKM plots of the difference spectrum shown in Figure S7c. KMD plots constructed from (a) positive and (b) negative peaks of the difference spectrum. RKM plots constructed from (c) positive and (d) negative peaks of the difference spectrum.

7. Verification of the reproducibility of EGA-MS measurements

To confirm the reproducibility of EGA-TOFMS measurements, ND/MASEBS was measured twice. Figure S9 shows a comparison of the results obtained from two EGA-TOFMS measurements of ND/MASEBS. It is evident that the TIC curves and temperature-dependent mass spectra remain nearly unchanged between two measurements.

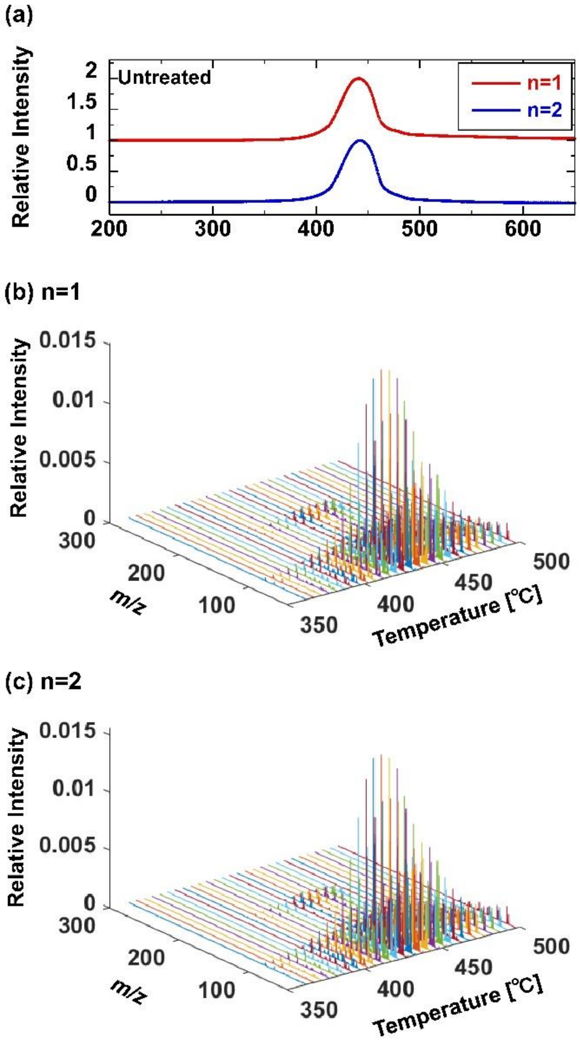

Figure S9. A comparison of the results obtained from two EGA-TOFMS measurements of ND/MASEBS; (a) TIC curves, temperature-dependent mass spectra obtained during the (b) first and (c) second EGA-TOFMS measurements.

REFERENCES

(1) Shieh, Y. T.; Wang, W. W. Radical scavenging efficiencies of modified and microwave-treated multiwalled carbon nanotubes. *Carbon* **2014**, *79*, 354-362.

(2) Shi, X.; Jiang, B.; Wang, J.; Yang, Y. Influence of wall number and surface functionalization of carbon nanotubes on their antioxidant behavior in high density polyethylene. *Carbon* **2012**, *50*, 1005-1013.

(3) Watanabe, R.; Oishi, A.; Nakamura, S.; Hagihara, H.; Shinzawa, H. Real-time monitoring of the thermooxidative degradation behavior of poly(acrylonitrile-butadiene-styrene) using isothermal in-situ Fourier transform infrared spectroscopy combined with principal component analysis. *Polymer* **2023**, *283*, 126243.

(4) Socrates, G. *Infrared and raman chacteristic group frequencies: Tables and charts,* 3rd Edition.; John Wiley and Sons: New York, 2004.
